# Genome-Wide Characterization of *Chrysanthemum indicum Nuclear Factor Y, Subunit C* Gene Family Reveals the Roles of *CiNF-YCs* in Flowering Regulation

**DOI:** 10.3390/ijms232112812

**Published:** 2022-10-24

**Authors:** Xueting Wang, Yao Yao, Shiyun Wen, Jing Bin, Qinghua Tan, Jinpeng Lou, Li Xie, Ruizhen Zeng, Herong Guo, Zhisheng Zhang, Qian Wei

**Affiliations:** 1 Guangdong Key Laboratory for Innovative Development and Utilization of Forest Plant Germplasm, College of Forestry and Landscape Architecture, South China Agricultural University, Guangzhou 510642, China; 2 College of Horticulture, South China Agricultural University, Guangzhou 510642, China

**Keywords:** nuclear factor Y, subunit C, transcription factor, expression, flowering regulation, chrysanthemum

## Abstract

Nuclear Factor Y, Subunit C (NF-YC) transcription factors are conserved in most plants, and play essential roles in plant growth and development, especially in flowering regulation. Chrysanthemums are important commercial plants, and their market value is strongly impacted by flowering time. Until now, no details regarding the NF-YC family in the *Chrysanthemum* genus have been available. In this study, five *NF-YC* genes were cloned from *Chrysanthemum indicum*. Multiple alignments showed that CiNF-YCs had the highly conserved characteristic regions. Phylogenetic analyses identified a pair of paralogue NF-YC proteins in chrysanthemums. Gene structure and conserved motifs were also analyzed for functional understanding. According to the results of the expression experiments, *CiNF-YC1* and *CiNF-YC5* were mainly expressed in leaves or flowers, and their expression levels varied greatly from the seedling to flower bud differentiation stage. *Arabidopsis* overexpressing *CiNF-YC1* and *CiNF-YC3* showed significantly delayed flowering, accompanied by other morphological alterations. RT-qPCR analysis revealed that genes associated with photoperiod, vernalization, aging, and gibberellin pathways were downregulated in *CiNF-YC1*-OX lines, relative to the wild type, whereas in *CiNF-YC3*-OX lines, only *SHORT VEGETATIVE PHASE* (*AtSVP*)*,* the key factor in the ambient temperature pathway, was upregulated. Taken together, these findings suggest that *CiNF-YC1* and *CiNF-YC3* negatively regulate flowering in *Arabidopsis* via different flowering pathways.

## 1. Introduction

Flowering is a major developmental transition in the life cycle of all angiosperms, and is determined by complex internal and external factors [1]. Many plants have evolved several mechanisms that control this fundamental process. Previous studies on *Arabidopsis thaliana* have confirmed that flowering is controlled by five major pathways: photoperiod, vernalization, gibberellin (GA), autonomous, and aging pathways [2]. These pathways interact with each other to form a complex regulatory network, and converge on a common set of floral integrators, such as *FLOWERING LOCUS T* (*FT*) and *SUPPRESSOR OF OVEREXPRESSION OF CO1* (*SOC1*), and ultimately induce the floral meristem identify genes, including *APETALA1* (*AP1*), *LEAFY* (*LFY*), and so on [2,3,4].

Among these flowering pathways, the photoperiod and vernalization pathways control flowering in response to seasonal changes in day length and temperature [2]. CONSTANS is a conserved central component of the photoperiod pathway. In *Arabidopsis*, CO integrates circadian clock and light signals, and promotes flowering by initiating transcription of the *FT* [4]. FLOWERING LOCUS C (FLC) is a central component of the vernalization pathway that acts as a potent repressor of flowering. Cold temperature triggers the silencing of *FLC*, subsequently promoting the transcriptional activation of *SOC1* and *FT* [2]. The GA pathway induces flowering mainly through bioactive GA, and some key enzymes have been proved to play key roles in flowering, such as GIBBERELLIN 20 OXIDASE (GA20ox), GIBBERELLIN 3 OXIDASE (GA20ox), and GIBBERELLIN 2 OXIDASE (GA2ox). The ambient temperature pathway responds to daily growth temperatures [2]. *Arabidopsis* plants flower earlier when grown at higher temperatures than at lower temperatures. SHORT VEGETATIVE PHASE (SVP), a MADS box transcription factor, appears to play a crucial role in this pathway. SVP increases the transcript level of *FT* at higher temperatures, but represses *FT* transcription at lower temperatures. The aging pathway provides an endogenous developmental cue that prevents flowering during the juvenile phase, and ensures flowering during the adult phase [5]. MicroRNA 156 (miR156) and SQUAMOSA PROMOTER BINDING PROTEIN-LIKE (SPL) have been identified as key modules of the aging pathway. As the plant ages, the concentration of *miR156* decreases, and *SPLs* increase. SPLs promote flowering through activating the expression of floral integrator, such as *LFY*, *FRUITFULL* (*FUL*), and *SOC1*.

More and more studies have proved that the nuclear factor Y (NF-Y) is also involved in flowering time regulation [5,6,7,8]. NF-Y is a heterotrimeric transcription factor complex that consists of three distinct proteins, NF-YA, NF-YB, and NF-YC [9]. In mammals, NF-YB and NF-YC subunits initially form a heterodimer in the cytoplasm, and then transfer to the nucleus to recruit the NF-YA proteins to form a complete NF-Y complex, which controls the expression of target genes [10]. In the plant lineage, only a few NF-Y complexes controlling the expression of a particular gene or process have been described [11,12]. Instead, single NF-Y subunit genes have been found to be involved in many developmental processes, including drought resistance, ABA signaling, embryogenesis, nitrogen fixation nodule development, flowering time, and so on [13,14,15,16,17,18].

Nuclear Factor Y, Subunit C (NF-YC), also known as Histone-Associated Protein 5 (HAP5) or CCAAT Binding Factor C (CBF-C), is a subunit of NF-Y trimers [19]. The NF-YC gene family is considered to participate in flowering time regulation [7,20,21,22,23,24]. In *Arabidopsis*, NF–YC3, NF–YC4, and NF–YC9 have high amino acid identity. Under long day (LD) conditions, all three *nf-yc* double mutants showed a mild delay in flowering, whereas the *nf-yc* triple mutant had significantly delayed flowering. Furthermore, an overexpression of *CONSTANS* (*CO*), the core member of the photoperiodic pathway, in the *nf-yc3/nf-yc4/nf-yc9* mutant, still could not restore a normal flowering time [22]. AtNF-YC3, AtNF-YC4, and AtNF-YC9 proteins can interact with AtNF-YB2, AtNF-YB3, and CO to form CO/AtNF-YB/AtNF-YC trimeric complexes, and then bind the proximal CCAAT motif in the *FT* promoter [7]. In rice, OsNF-YC2/4/6 were identified as the homologous proteins of AtNF-YC3/4/9, and were associated with flowering time regulation [7,21]. Among them, OsNF-YC2 and OsNF-YC4 inhibited flowering under LD conditions, whereas OsNF-YC6 expression induced flowering. In *Arabidopsis*, the heterologous overexpression of *ZmNF-YC14* delayed flowering and significantly changed the morphology of the inflorescence [25]. These results suggested that the flowering time regulation of NF-YC was conservative in different species, but had functional redundancy.

Chrysanthemums are ornamental plants with important commercial value, as well as important health foods and anti-inflammatory traditional Chinese medicines [26]. Their commercial value is largely attributed to the blooming chrysanthemum plants. Therefore, it is crucial to understand the molecular mechanisms underlying the time to blooming. Previous studies have shown that NF-YC is a key factor in regulating flowering time, but the function of NF-YC in chrysanthemums has not been reported. In this study, we identified five NF-YC family genes in wild chrysanthemums (*Chrysanthemum indicum*), and investigated the characteristics, phylogenetic relationships, gene structures, and conserved motifs of these genes. We also analyzed the expression patterns of *CiNF-YC* genes in different tissues and different developmental stages. In addition, we showed that *CiNF-YC1* and *CiNF-YC3* negatively regulated the flowering time when overexpressed in *Arabidopsis*. These results will provide a good basis for the further functional characteristics of *CiNF-YC* genes and their utilization for the genetic improvement of chrysanthemums.

## 2. Results

### 2.1. Identification of the NF-YC Family Members

**Figure 1:**
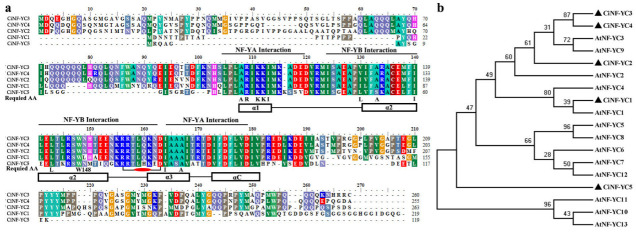
Multiple alignment and phylogenetic analysis for wild chrysanthemum NF-YC proteins. (**a**) Multiple alignments of full length of CiNF-YC proteins. The subunit interaction domain is represented. The alpha-helices (rectangles) and coils (black lines) are marked on the bottom. The required amino acids (required AA) are given on the bottom of the alignment. Conserved typtophan (W148) and the putative arginine (R)-aspartate (D) pairs were marked. (**b**) Phylogenetic analysis of NF-YC proteins in *Arabidopsis thaliana* and *Chrysanthemum indicum* (▲).

In order to identify NF-YC family members in wild chrysanthemums, the conserved NF-YC domains from *Arabidopsis* were used to screen for NF-YCs in the *Chrysanthemum nankingense* genome, resulting in the identification of five unique nucleotide sequences. Using the obtained *CmNF-YC* sequences as references, primers were designed, and five *CiNF-YCs* were amplified by PCR in *Chrysanthemum indicum* (Data S1, Figure S2). These CiNF-YCs were named according to the closest homologues of them in *Arabidopsis* (Figure 1b and Figure S1). Detailed information is listed in Table 1. The gene lengths of *CiNF-YCs* ranged from 360 bp to 796 bp, and the CiNF-YC proteins ranged from 120 aa to 264 aa in length, whereas the corresponding protein molecular weights (MWs) ranged from 13.10 kDa to 29.44 kDa. The predicted theoretical isoelectric points (pI) ranged from 4.82 to 6.89, indicating that all NF-YC proteins were located in acidic environments, such as the nucleus.

### 2.2. Multiple Alignment and Phylogenetic Analysis of NF-YC Proteins in Wild Chrysanthemum

Multiple alignments of the deduced amino acid sequences of CiNF-YCs were performed (Figure 1a). All CiNF-YC proteins had structural similarities with H2A histone, and contained conserved NF-YA/B interaction domains. Most required amino acids were highly conserved in all CiNF-YCs proteins (Figure 1a). The characteristic intra-chain arginine-aspartate (R156-D163) bidentate pair, which was required for the stability of the NF-YB/C heterodimer, was completely conserved. Another specific feature, tryptophan (W148) at the end of α2, which allowed the specific interaction of NF-YC and NF-YB, was also completely conserved in all CiNF-YCs. The highly conserved peptide motifs suggested that all CiNF-YCs retain the ability to bind to NF-YB in evolution.

To reveal the phylogenetic relationship and potential function of the CiNF-YCs, an unrooted phylogenetic tree was generated with the neighbor-joining (NJ) method using the full length of NF-YC proteins identified in wild chrysanthemums and *Arabidopsis* (Figure 1b). CiNF-YC1 was the closest homologue to AtNF-YC1. A pair of paralogues, CiNF-YC3 and CiNF-YC4, showed high similarity in sequence, and were the homologues of AtNF-YC3 and AtNF-YC9 (Figure 1b).

### 2.3. Gene Structure and Conserved Motif Analyses of NF-YC in Wild Chrysanthemum

**Figure 2:**
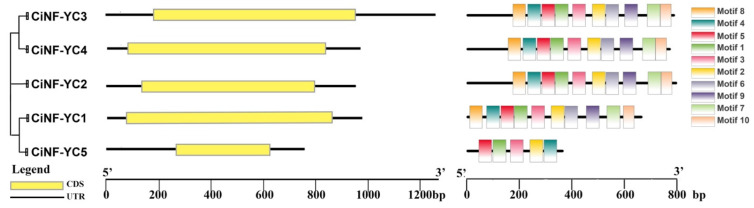
The exon-intron structure and conserved motifs of NF-YC ORF region in wild chrysanthemums. Exons and UTRs are indicated with yellow rectangles and black lines, respectively. Each conserved motif is represented by a number of colored rectangles. Box length corresponds to motif length. Motif 1: AAGATCATGAARGCAGAYGAGGAYGTDMGVATGATATCAGCTGARGCACC; Motif 2: CAVAAAAACGAYATTGCWGCTGCAATYACRAGGACTGATATHTTTGAYTT; Motif 3: GCATGTGARAT GTTCATHCTDGAGTTGACTHTAMGGTCATGGAATCATAC; Motif 4: CADCAAYTDCATCAACAACTBC ARAADTTTTGGGMNAACCARTRYCRAGA; Motif 5: GARMAADYNAVTGATTTYAARAACCATAGNYT NCCATTRGCAAGDATHAA; Motif 6: TDGTTGATATTGTWCCAAGDGAGGATHTDAAAGATGADGTTV TBGCWTC; Motif 7: GCCTGTGGATCCTCMDGSTCTTTATGGACAGCAGCCTCVDCCATABATGG; Motif 8: CAGCACAACTHGCACAACAGCAACTBGCTTATCABCACATHCAHCAVC; Motif 9: CTBCCAGTTGGHG GBCCWACTGAKRBBCTCCCDTABTAYTACATGCCDCC; Motif 10: STCMRCCWMTBTGGCCACAVCMRC ADCAVCAABMACCVCMGG.

To predict the function of the *CiNF-YC* genes, the exon-intron structures of *CiNF-YCs* and the conserved motifs in the ORF (Opening Reading Frame) region of *CiNF-YCs* were analyzed (Figure 2). Based on the genome sequences and corresponding coding sequences of *CiNF-YC* genes, we found that no *CiNF-YCs* contained introns. The MEME analysis discovered 10 putative motifs in total, namely motif 1 to motif 10 (Supplementary Figure S3). Motif 1–5 were shared by all *CiNF-YCs*, indicating that these conserved motifs were essential for group-specific functions. Except for *CiNF-YC5*, the majority of *CiNF-YCs* featured motifs 6–10. These results suggested that *CiNF-YC5* might have different functions from other *CiNF-YCs*.

### 2.4. Expression Profiles of CiNF-YC Genes

**Figure 3:**
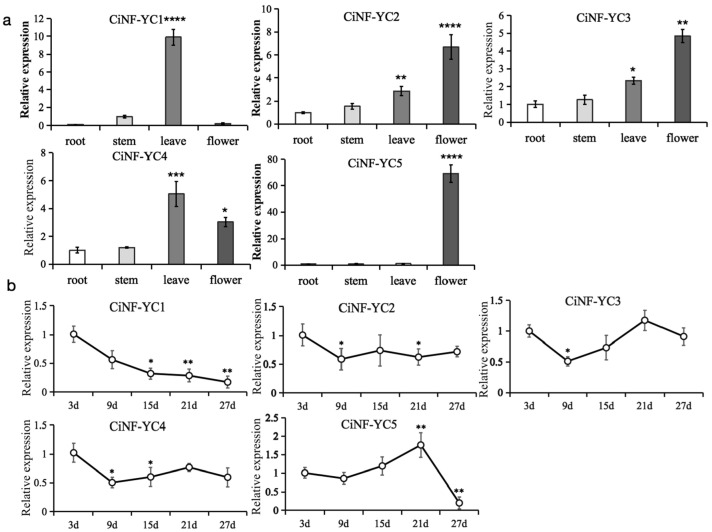
Transcript abundance of *CiNF-YCs* in different tissues and developmental stages. (**a**) Transcript abundance of *CiNF-YCs* in different tissues. After 52 days of induction under SD conditions, the plants at full flowering stage were sampled. RT-qPCR was performed to evaluate expression of *CiNF-YCs*, using *Actin* (GenBank accession AB770470) as the control. For *CiNF-YC1*, each tissue was compared to the stem. For other *CiNF-YC* genes, each tissue was compared to the root. (**b**) Transcript abundance of *CiNF-YCs* in leaves of differently aged wild chrysanthemums. Plants were grown under SD conditions. Samples were harvested every 6 days from 3 days after propagation. RT-qPCR was performed, and each time point was compared to 3d. Three independent experiments were performed, and error bars indicate standard deviation. * *p* < 0.05, ** *p* < 0.01, *** *p* < 0.001, and **** *p* < 0.0001 indicate significant differences.

To investigate the biological roles of CiNF-YCs in wild chrysanthemums, the expression profiles of *CiNF-YC* genes were evaluated in different organs (leaf, stem, root, and flower) of the plants under short day (SD) conditions. As shown in Figure 3a, the transcript abundance of *CiNF-YC1* in leaves was 10 times higher compared to other organs. The expression level of *CiNF-YC5* in flowers was significantly higher than that in other organs. *CiNF-YC3* and *CiNF-YC2* had the highest expression in leaves, followed by flowers. The expression of *CiNF-YC4* was the highest in flowers, followed by leaves.

To further evaluate the functions of CiNF-YCs in floral transition, we measured the expression level of the CiNF-YCs every 6 days in the leaves of plants grown in SD until the plants flowered (day 27). The expression of *CiNF-YC1* peaked in young seedlings, and subsequently declined with development (Figure 3b), indicating that *CiNF-YC1* might be a negative factor in floral transition. Both *CiNF-YC3* and *CiNF-YC4* started to decrease on the ninth day, and then return to the initial level detected at 3d. The similar change trend suggested that CiNF-YC3 and CiNF-YC4 might have functional redundancy. The expression of *CiNF-YC5* was increased sharply on the 21st day, and then rapidly decreased to a very low level, implying that it might play a role in flower development. The expression of *CiNF-YC2* remained consistent from day 9 to day 27. Taken together, the expression of *CiNF-YC1* decreased continuously from the seedling stage to flowering stage, and the expression of *CiNF-YC5* changed significantly before flowering, whereas *CiNF-YC2*, *3*, and *4* changed mildly throughout the development stage. These results indicate that *CiNF-YC1* might play an important role in flower induction, and *CiNF-YC5* might be related to flower organ development.

### 2.5. Modified Flowering Time of CiNF-YC1 Overexpressing Arabidopsis

**Figure 4:**
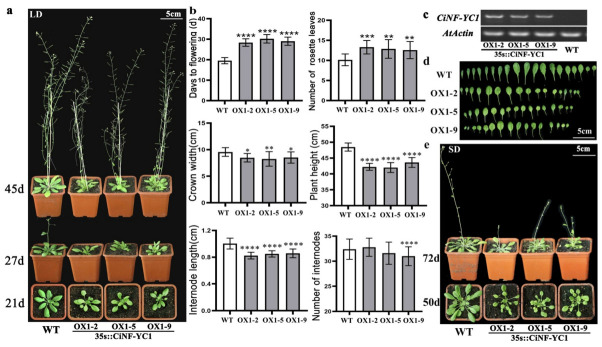
Flowering time and phenotype analysis of *CiNF-YC1* overexpressing *Arabidopsis*. (**a**) Flowering time of *CiNF-YC1* overexpressing *Arabidopsis* under LD conditions. (**b**) Phenotype analysis of *CiNF-YC1* overexpressing *Arabidopsis* under LD conditions. Ten independent plants were measured, and error bars indicate standard deviation. * *p* < 0.05, ** *p* < 0.01, *** *p* < 0.001, and **** *p* < 0.0001 indicate significant differences. (**c**) Identification of *CiNF-YC1* overexpressing *Arabidopsis* by RT-PCR. (**d**) The leaf size of WT plants and *CiNF-YC1* overexpressing *Arabidopsis*. (**e**) Flowering time of *CiNF-YC1* overexpressing *Arabidopsis* under SD conditions.

According to the results of gene expression in different tissues and different development stages, *CiNF-YC1* was selected to validate its relationship with flowering. We generated transgenic *Arabidopsis* homozygous lines overexpressing *CiNF-YC1* under the control of the CaMV35S promoter. After verification of the transcript presence of *CiNF-YC1* using RT-PCR, three *CiNF-YC1*-OX lines (OX1-2, OX1-5, and OX1-9) were selected for further analysis (Figure 4c). Compared to WT plants, the *CiNF-YC1*-OX plants significantly delayed flowering under LD conditions, and the differences were recorded at 21d, 27d, and 45d, respectively (Figure 4a). At 20 days, flower bud emergence was observed in WT plants, whereas it was not observed in *CiNF-YC1*-OX lines 2, 5, or 9 until days 28, 30, and 29, respectively (Figure 4b). At 27 days, the number of rosette leaves in WT plants was 10, whereas that of the *CiNF-YC1*-OX lines was 13 (Figure 4b). To further determine whether the delayed flowering caused by *CiNF-YC1* was dependent on day length, the flowering time of *CiNF-YC1*-OX plants under SD conditions was also observed. Under SD conditions, *CiNF-YC1*-OX plants still displayed the late flowering phenotype, and the differences were recorded at 50 d and 72 d, respectively (Figure 4e).

In addition, *CiNF-YC1*-OX plants also displayed other morphological alterations, such as smaller leaves, decreased crown width, and shorter plant height (Figure 4b,d). The height of WT plants was 48.5 cm and the height of *CiNF-YC1*-OX lines was 42–44 cm. The plant height mainly depends on the number and length of internodes. Compared with WT plants, *CiNF-YC1*-OX *Arabidopsis* plants showed an obvious decrease in the internode length. Taken together, the results indicated that the negative regulation of flowering time by *CiNF-YC1* did not depend on the day length, and might be related to development.

### 2.6. Effects of CiNF-YC3 on Flowering and Growth of Arabidopsis

**Figure 5:**
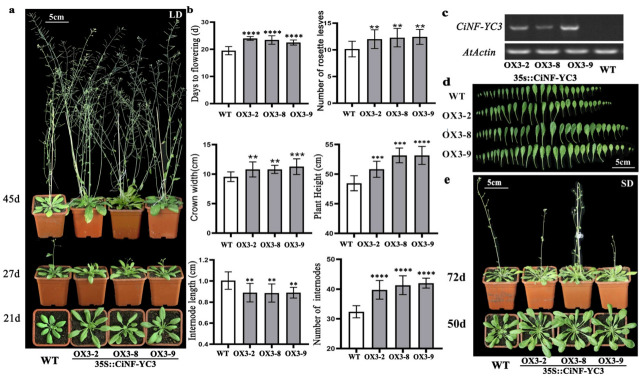
Flowering time and phenotype analysis of *CiNF-YC3* overexpressing *Arabidopsis*. (**a**) Flowering time of *CiNF-YC3* overexpressing *Arabidopsis* under LD conditions. (**b**) Phenotype analysis of *CiNF-YC3* overexpressing *Arabidopsis* under LD conditions. Fifteen independent plants were measured, and error bars indicate standard deviation. ** *p* < 0.01, *** *p* < 0.001, and **** *p* < 0.0001 indicate significant differences. (**c**) Identification of *CiNF-YC3* overexpressing *Arabidopsis* by RT-PCR. (**d**) The leaf size of WT plants and *CiNF-YC3* overexpressing *Arabidopsis*. (**e**) Flowering time of *CiNF-YC3* overexpressing *Arabidopsis* under SD conditions.

It has been reported that AtNF-YC3 and AtNF-YC9 positively regulate flowering time under LD conditions. *CiNF-YC3* and *CiNF-YC4* are the homologue genes of *AtNF-YC3* and *AtNF-YC9*, and *CiNF-YC3* was chosen to further investigate its role in flowering. We obtained ten lines of *CiNF-YC3* overexpressing *Arabidopsis*, and three homozygous lines (OX3-2, OX3-8, and OX3-9) were confirmed by RT-PCR and selected for further analysis (Figure 5c). *CiNF-YC3*-OX plants flowered 2–3 days later than WT plants under LD conditions (Figure 5b), and the delayed flowering was also observed under SD conditions (Figure 5e). In addition, *CiNF-YC3*-OX plants also displayed other morphological alterations, such as increased leaf size, crown width, and plant height (Figure 4b,d). The height of WT plants was 48.5 cm, and the height of *CiNF-YC3*-OX lines was 50.8–53.5 cm. Compared with WT, *CiNF-YC3*-OX plants showed an increase in the internodes number and a decrease in internode length. These results indicate that *CiNF-YC3* plays a role in flowering and plant growth, but the regulatory mechanism is different from that of *CiNF-YC1*.

### 2.7. Modified Expression of Genes Involved in Flowering in CiNF-YC1 and CiNF-YC3 Overexpressor Lines

To investigate the regulation pathway of *CiNF-YCs*, RT-qPCR was performed to analyze the transcript abundance of key genes in five major flowering pathways, including *AtSVP* (ambient temperature pathway), *AtCO* (photoperiod pathway), *AtSPLs* (age pathway), *AtGA20oxs* (GA pathway), and *AtFLC* (vernalization pathway). Relative to WT plants, the expression of *AtCO, AtSPL3*, *AtSPL4*, *AtGA20ox3*, and *AtGA20ox4* was clearly downregulated in the *CiNF-YC1*-OX lines, whereas the expression of *AtFLC* was dramatically upregulated. The expression of *AtSPL5*, *AtSVP*, *AtGA20ox1*, and *AtGA20ox2* showed no obvious differences between *CiNF-YC1*-OX *Arabidopsis*, and WT plants. In *CiNF-YC3*-OX lines, only *AtSVP*, the key gene in the ambient temperature pathway, was upregulated. In addition, the expression of flowering integrators, *AtFT*, *AtLEAFY*, and *AtSOC1*, was significantly decreased in *CiNF-YC1*-OX lines. However, in *CiNF-YC3*-OX lines, only the expression of *AtFT* was significantly decreased.

These results suggest that the influence of *CiNF-YC1* on flowering time may be related to multiple flowering pathways directly or indirectly, whereas *CiNF-YC3* has a close relationship with the ambient temperature pathway.

## 3. Discussion

### 3.1. Characterization of the NF-YC Gene Family in Chrysanthemum Indicum

NF-Y is a heterotrimeric transcription factor that includes three subunits: NF-YA, NF-YB, and NF-YC, and is conserved in nearly all eukaryotes. In the past two decades, many genes encoding NF-YC proteins have been identified in several species, including 20 *NF-YC* genes in *Solanum lycopersicum* [27], 15 *NF-YC* genes in *Glycine max* [28], 14 *NF-YC* genes *Triticum aestivum* [29], 14 *NF-YC* genes in *Sorghum bicolor* (L.) *Moench* [30], 14 *NF-YC* genes in *Populus tomentosa* [31], 13 *NF-YC* genes *Arabidopsis thaliana* [32], 13 *NF-YC* genes in *Setaria italica* [33], 12 *NF-YC* genes in *Brachypodium distachyon* [34], 12 *NF-YC* genes in *Physcomitrella patens* [35], 10 *NF-YC* genes in *Malus Domestica* [36], 9 *NF-YC* genes in *Arachis Hypogaea* L. [37], 9 NF-YC genes in *Vitis vinifera* [38], 7 *NF-YC* genes in *Phaseolus vulgaris* [39], 7 *NF-YC* genes in *Z. jujube* [40], 7 *NF-YC* genes in *Oryza sativa* [41], 6 *NF-YC* genes in *Prunus persica* [42], 5 *NF-YC* genes in *Citrus* [43], 4 *NF-YC* genes in *Citrullus lanatus* [44], 4 *NF-YC* genes in *Petunia hybrida* [45], and 3 *NF-YC* genes in *Brassica napus* [46]. In this study, five *NF-YC* coding genes were obtained by PCR amplification in wild chrysanthemums, which was less than that reported in most plants. This might be explained by a contraction of the NF-YC family in wild chrysanthemums, as well as by the limitation of reference genome data used in the study [47]. Notably, CiNF-YC5 was different from other members in several aspects (Table 1), including protein length, molecular weight, theoretical isoelectric point, and expression pattern, which suggested the unique functions of CiNF-YC5.

In general, proteins can be classified by their conserved characteristic regions. Multiple alignments showed that the characteristic region of the NF-YC family was highly conserved in all CiNF-YC proteins, and the required amino acids previously reported in mammals were also conserved (Figure 1a) [32,38]. This high conservation suggested that CiNF-YCs retained functions related to the complex formation in mammals. Notably, the amino acid sequences of CiNF-YC3 and CiNF-YC4 were highly consistent, especially in the conserved region (Figure 1a). Moreover, phylogenetic analysis also indicated that *CiNF-YC3* and *CiNF-YC4* were a pair of paralogues and had homologous genes in *Arabidopsis* (Figure 1b). Therefore, the function of CiNF-YC 3 and 4 can be predicted according to the function of their homologous genes in *Arabidopsis*. In addition, CiNF-YCs were absent on several branches, and only AtNF-YCs were presented.

Gene organization plays an important role in the evolution of gene families, and is also an important basis for predicting gene function. Gene structural analysis of *CiNF-YC* gene families showed that all *CiNF-YC* genes lacked introns (Figure 2). The result was consistent with the gene structure of *MdNF-YC* and *StNF-YC* families [36,48]. Intriguingly, a few *NF-YC* members in other reported plant species had introns, such as poplar [31], sorghum [30], common bean [39], and Foxtail Millet [49]. Previous studies have reported that introns were ubiquitous in eukaryotes and had many functions, such as alternative splicing, the reduction of gene damage, and the regulation of gene expression [48,50,51,52]. In general, alternative splicing always led to the rapid expansion of genes. The lack of the intron of *CiNF-YCs* may limit the expansion of the CiNF-YC family in evolution.

Another key piece of evidence related to the gene family’s function is the variety of motifs. According to the motif analysis of *CiNF-YCs*, motif 1–3 functioned as DNA interaction, and motif 4–7 functioned by interacting with others (Figure 2). The motif patterns of *CiNF-YCs* were associated with their phylogenetic relationship. These findings suggested that CiNF-YCs belonging to the same clade may have a similar function.

### 3.2. Expression Patterns of CiNF-YC Genes in Various Developmental Stages and Tissues

The functions of NF-YC genes have been well investigated in model plants, demonstrating involvement in flowering time [20,21,22,24], abiotic response [14,18,20,53], hormone response [54,55,56], and plant growth and development [54,55,57,58]. However, their roles in chrysanthemums remain unclear.

It was shown that *CiNF-YC2*, *3*, and *4* had relatively higher expression levels in flowers and leaves, suggesting that they were involved in flower or leaf development. It was possible that these three members had functional redundancy in evolution. The highest expression levels of *CiNF-YC1* and *CiNF-YC5*, which were dozens of times higher than those in other tissues, were found in leaves or flowers, respectively. This suggested that *CiNF-YC1* and *CiNF-YC5* might be essential and irreplaceable to the development of leaves or flowers. Previous studies have confirmed that NF-YCs regulated flowering time mainly through the photoperiodic pathway. To investigate the roles of *CiNF-YCs* in flowering, their expression was analyzed from the young seedling stage (three days after propagation) to the flower bud differentiation stage (27 days after propagation). It is widely known that the leaf is the major organ for flowering signal transduction. Combined with the results of tissue-specific expression, the leaf was chosen for the expression experiments. Interestingly, *CiNF-YC2*, *CiNF-YC3*, and *CiNF-YC4* maintained high expression levels before flower bud differentiation. One explanation was that they played diverse functions at various developmental stages through forming various NF-Y trimers with NF-YA and NF-YB subunits. The expression of *CiNF-YC1* continued to decrease before day 27, indicating that it might play a role in flowering time regulation. *CiNF-YC1* was closely placed with *AtNF-YC1* and *AtNF-YC4*, which were known to associated with the regulation of flowering [22]. These data led us to the conclusion that *CiNF-YC1* might play a negative role in flowering. *CiNF-YC5* peaked on day 21 and then decreased on day 27. However, *CiNF-YC5* had no close relatives in *Arabidopsis*, and it was difficult to predict its function. The expression data indicated that it might be related to flowering or flower organ development.

### 3.3. Function of CiNF-YC Genes in Flowering

The roles of several *NF-YC* genes in flowering have been well investigated in *Arabidopsis* and rice. *AtNF-YC3*, *AtNF-YC4*, *AtNF-YC9*, and their homologous genes in rice had conserve functions, i.e., regulating flowering time via the photoperiodic pathway [21,22]. Notably, *OsNF-YC2* and *OsNF-YC4* acted as inhibitors of flowering under long-day conditions, whereas *OsNF-YC6* acted as the opposite [21], indicating that the NF-YC family had functional differentiation. In the present study, both *CiNF-YC1*-OX and *CiNF-YC3*-OX plants delayed flowering to varying degrees (Figure 4 and Figure 5). These findings were contrary to the functions of *AtNF-YC3*, *AtNF-YC4*, and *AtNF-YC9*, but were consistent with *ZmNF-YC14* [25]. Additionally, we noticed additional phenotypic alterations in *CiNF-YC1*-OX- and *CiNF-YC3*-OX-overexpressing plants, including changes in leaf size, plant height, crown width, internode length, and internode number (Figure 4b,d and Figure 5b,d). Similarly, *nf-yc3/4/9* displayed a decreased crown width compared to WT [22]. Increased plant height was observed in *ZmNF-YC14*-overexpressing *Arabidopsis* [25]. These data revealed that NF-YCs were involved in flowering time regulation, and also participated in plant growth and development. The mechanisms by which NF-YC regulates plant growth and development will be an interesting question in the future.

### 3.4. Possible Flowering Pathways That Involved by CiNF-YC1 and CiNF-YC3

It was first reported in *Arabidopsis* that NF-YC played a critical role in the photoperiodic pathway by interacting directly with CONSTANS (CO) [20,22]. Subsequently, the involvement of NF-YC in the photoperiodic flowering response was reported in rice [21], maize [25], poplar [23], and other plants. Recent studies have shown that the regulation of flowering by NF-Y family members may also be related to the GA and age pathway [7,25]. To explore the flowering pathways that *CiNF-YC1* and *CiNF-YC3* participated in, we detected the expression levels of key genes involved in five major flowering pathways.

**Figure 6:**
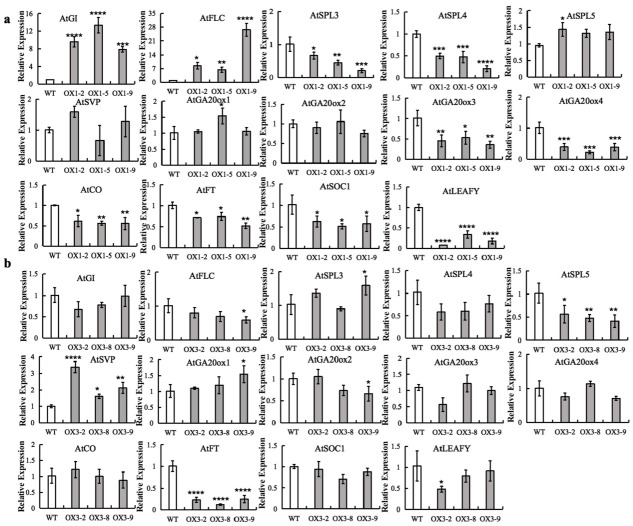
Transcript abundance of flowering-related genes in *CiNF-YC1*- and *CiNF-YC3*-overexpressing *Arabidopsis*. (**a**) Transcript abundance of flowering-related genes in *CiNF-YC1*-overexpressing *Arabidopsis* under LD conditions. (**b**) Transcript abundance of flowering-related genes in *CiNF-YC3*-overexpressing *Arabidopsis* under LD conditions. Three independent experiments were performed, and error bars indicate standard deviation. * *p* < 0.05, ** *p* < 0.01, *** *p* < 0.001, and **** *p* < 0.0001 indicate significant differences.

In the *CiNF-YC1*-OX *Arabidopsis*, the expression of *AtCO, AtSPL3*, *AtSPL4*, *AtGA20ox3*, and *AtGA20ox4* was clearly downregulated, whereas the expression of *AtFLC* was dramatically upregulated (Figure 6a). These results indicated that CiNF-YC1 participated in multiple flowering pathways, including the photoperiod pathway, vernalization pathway, age pathway, and GA pathway. A previous study showed that GA treatment could partially rescue the delayed flowering of *ZmNF-YC14*-overexpressing plants [25]. Another study showed that *CmNF-YB8* negatively regulated flowering time through the aging pathway in chrysanthemums [7], but the NF-YCs with which it formed a trimer were still unclear. In previous studies, the histone methyltransferase CURLY LEAF (CLF) could mediate H3K27me3 deposition in FT in the photoperiodic pathway, and indirectly controls *FT* expression by epigenetically regulating FLC [24]. In *Arabidopsis*, NF-YC could bind to the CLF, and relieved the repression of *FT* transcription under LD conditions. However, whether NF-YC can epigenetically regulate FLC requires further investigation.

In the *CiNF-YC3*-OX lines, the expression of *AtSVP* was clearly upregulated. Until now, there has been no relevant report on the involvement of NF-YC in the ambient temperature pathway. Interestingly, *AtSPL5* was downregulated in *CiNF-YC3*-OX plants and upregulated in *CiNF-YC1*-OX plants. In *Arabidopsis*, *SPLs* were proved to modulate leaf morphology [59]. We concluded that *AtSPL5* might be connected to changes in crown width and leaf size.

This evidence indicates that NF-YC members participate in flowering regulation directly or indirectly, but the regulatory mechanisms are not the same, which may be connected to the diversity of NF-Y heterotrimers.

## 4. Materials and Methods

### 4.1. Identification, Alignments, and Phylogenetic Analysis of NF-YCs in Wild Chrysanthemum

The coding and protein sequences of AtNF-YCs were retrieved from the Arabidopsis Information Resource (TAIR, https://www.arabidopsis.org/) (accessed on 10 September 2020), and then the conserved sequences were utilized as queries to search the protein and nucleotide sequences of CmNF-YCs in the chrysanthemum genome database (http://www.amwayabrc.com) (accessed on 7 October 2020). SMART (http://smart.embl-heidelberg.de/) (accessed on 10 October 2020) was used to check for the presence of the NF-YC domain in the obtained sequences. The repeated sequences and sequences without the NF-YC domain were removed. Using the nucleotide sequences of CmNF-YCs as the reference sequences, the primers were designed to amplify the genome and coding sequences of *NF-YCs* in wild Chrysanthemums (Table S1).

The molecular weight (MW) and isoelectric point (pI) of the deduced amino acid sequences were predicted by the Prosite ExPASy server (http://web.expasy.org/protparam/) (accessed on 7 June 2021). The alignments of full-length amino acid sequences of CiNF-YCs and AtNF-YCs were performed using ClustalX (http://www.clustal.org/) (accessed on 4 January 2021) and BioEdit. A neighbor-joining tree was constructed using MEGA6 with the following parameters: model, JTT + G + F substitution; bootstrap replicates, 1000 [60].

### 4.2. Gene Structure and Motif Analyses of NF-YCs in Wild Chrysanthemum

The exons and introns of the *CiNF-YCs* were analyzed using Gene Structure Display Server 2.0 [61] (https://gsds.cbi.pku.edu.cn/) (accessed on 11 November 2021). The conserved motifs in the ORF region of *CiNF-YCs* were predicted using MEME (http://meme-suite.org/) (accessed on 11 November 2021) [62], with the following parameter settings: (1) width of optimum motif, greater than 6 and less than 100; (2) maximum number of motifs, 10; (3) e-value, less than 1 × 10^−10^. The gene structure and conserved motifs were visualized on the TBtools toolkit [63].

### 4.3. Plant Materials and Culture Conditions

The wild chrysanthemum (*Chrysanthemum indicum*) used in this study was propagated by in vitro culturing. Chrysanthemum shoots, including 1 node, were cultured on a medium comprising 1/2 Murashige and Skoog (MS) for 40 days in a culture room at 23 ± 1 °C, 40% relative humidity, and 100 μmol m^−2^ s^−1^ illumination with fluorescent lamps under a LD cycle (16 h light /8 h dark). Then, the plants were transplanted into 9 cm diameter pots containing a mixture of 1:1 (*v*/*v*) peat and pearlite, and grown in a culture room at 25/18 °C day/night, 60% relative humidity, and 250 μmol m^−2^ s^−1^ illumination with fluorescent lamps under a SD cycle (10 h light/14 h dark).

*Arabidopsis thaliana* ecotype Columbia (Col-0) was the genetic background of wild-type and transgenic lines used throughout the work. The plants were grown in a culture room at 25/18 °C day/night, 60% relative humidity, and 250 μmol m^−2^ s^−1^ illumination with fluorescent lamps under a LD cycle (16 h light/8 h dark) or SD cycle (10 h light/14 h dark).

### 4.4. RNA Isolation and RT-qPCR Analysis

For the tissue-specific transcript profiles of *CiNF-YCs*, samples of roots, stems, leaves, and flowers were harvested from the full flowering wild chrysanthemums. Obvious flower bud differentiation could be observed in most chrysanthemums after 27 days of SD treatment. In order to detect the expression patterns of *CiNF-YCs* before flowering, the second fully expanding leaves from the top were sampled at 3 d, 9 d, 15 d, 21 d, and 27 d after transplanting into the SD conditions. To evaluate the transcript profiles of flowering-related genes in *Arabidopsis*, the leaves of 14-day-old seedlings of WT and transgenic plants were sampled. After sampling, all of the collected materials were immediately frozen in liquid nitrogen and stored at −80 °C. Each treatment was replicated three times.

Total RNA was isolated from samples using an RNA Aprep Pure Plant Kit (Vazyme, Nanjing, China) according to the manufacturer’s instructions. The first cDNA strand was synthesized from 1 μg of total RNA using a HiScript^®^ III 1st Strand cDNA Synthesis Kit (+gDNA wiper) (Vazyme, Nanjing, China) according to the manufacturer’s instructions. RT-qPCR reactions (20 μL volume containing 1 μL cDNA as the template) were run using the CFX96 Real-Time PCR Detection System (Bio-Rad, CA, USA) in standard mode with the ChamQ Universal SYBR qPCR Master Mix Kit (Vazyme, Nanjing, China). Gene-specific primers were designed using the Primer-BLAST tool in NCBI (https://www.ncbi.nlm.nih.gov/tools/primer-blast/) (accessed on 10 October 2021), and the chrysanthemum *Actin* gene (GenBank accession AB770470) was used as an internal control. Relative transcript abundances were calculated via the 2^−ΔΔCt^ method. Three independent experiments were conducted. The primers for RT-qPCR are listed in Supplementary Table S1.

### 4.5. Generation of CiNF-YC1 and CiNF-YC3 Overexpressing Arabidopsis

To construct the *CiNF-YC1* and *CiNF-YC3* overexpression vector, the CDSs of *CiNF-YC1* and *CiNF-YC3* were amplified by primers containing restriction sites and homologous sequences of the vector, pCAMBIA1300. The PCR products were fused to the binary vector, pCAMBIA1300. The PCR primers are listed in Supplementary Table S1. The overexpression plasmids were transformed into *A. thaliana* using the floral dip method by *A. tumefaciens* strain GV3101. The transformants were screened on an MS basal medium containing 25 mg·L^−1^ Hygromycin B (Hyg), and confirmed by RT-PCR. T3 plants displaying 100% Hyg resistance were considered homozygous and were used in this study.

### 4.6. Functional Validation of CiNF-YC1 and CiNF-YC3 in Arabidopsis

Homozygous T3 *Arabidopsis* plants overexpressing the *CiNF-YC1* or *CiNF-YC3* were phenotypically characterized. The WT and Transgenic *Arabidopsis* plants were photographed at 21, 27, and 45 d after transplanting to soil. The number of rosette leaves and the crown width of the plants were measured at day 27. The plant height, internode number, and internode length were counted and measured after the pods matured. Three lines of *CiNF-YC1*-OX, *CiNF-YC3*-OX, and WT plants were selected, with five plants per line, and each experiment was repeated three times. All presented data were from the mean of 15 individual plants. The data were presented as the mean ± standard error of the mean, and were analyzed using GraphPad Prism version 8 (GraphPad Software Inc., San Diego, CA, USA). The error bars were calculated according to Tukey’s multiple range test, and with * *p* < 0.05, ** *p* < 0.01, *** *p* < 0.001, and **** *p* < 0.0001 being used to indicate statistically significant effects.

## 5. Conclusions

In this study, five *CiNF-YC* family genes were cloned from *Chrysanthemum indicum*. The characteristics, phylogenetic relationships, gene structures, and conserved motifs of CiNF-YCs were analyzed. The expression patterns of *CiNF-YC* genes in various tissues and developmental stages were also analyzed by RT-qPCR. Additionally, the negative roles of *CiNF-YC1* and *CiNF-YC3* in flowering regulation were demonstrated by comparing WT and *CiNF-YCs* overexpressing *Arabidopsis*. The regulatory mechanisms involved by *CiNF-YC1* and *CiNF-YC3* were preliminarily investigated through evaluating the expression of key genes in different flowering pathways. *CiNF-YC1* regulated flowering time through the photoperiod, GA, vernalization, and aging pathway directly or indirectly, whereas *CiNF-YC3* had a close relationship with the ambient temperature pathway. These results will provide a good basis for further functional analysis of *CiNF-YC* genes, and support their use in chrysanthemum genetic improvement.

## Figures and Tables

**Table 1:** Basic information of the *NF-YC* gene family in *chrysanthemum indicum*.

Gene Name	Genome ID	NCBI ID	mRNA Length (bp)	Peptide Residues (aa)	MW (kDa)	pI
*CiNF-YC1*	CHR00079409-RA	IABW01058102.1	659	220	23.89	4.82
*CiNF-YC2*	CHR00063225-RA	IABW01127607.1	796	264	29.44	5.27
*CiNF-YC3*	CHR00082598-RA	IABW01051895.1	786	261	29.13	6.89
*CiNF-YC4*	CHR00018499-RA	IABW01098859.1	768	256	28.66	5.04
*CiNF-YC5*	CHR00020456-RA	IABW01033460.1	360	120	13.10	6.80

pI, isoelectric point; MW, molecular weight.

## Data Availability

Not applicable.

## Supplementary Materials

The following supporting information can be downloaded at: https://www.mdpi.com/article/10.3390/ijms232112812/s1.
