# Supplementary material for: Genome-Wide Characterization of Chrysanthemum indicum Nuclear Factor Y, Subunit C Gene Family Reveals the Roles of CiNF-YCs in Flowering Regulation
Source: Int J Mol Sci. 2022 Oct 24;23(21):12812. doi: 10.3390/ijms232112812 (PMC9654237; doi:10.3390/ijms232112812)
Supplement: Supplementary file 1 [file ijms-23-12812-s001.zip › Supplemental Table S1. Primers used for gene isolation, vector construction and RT-qPCR analysis.pdf]

Supplementary Table S1. Primers used for gene isolation, vector construction and RT-qPCR analysis

Accession No.	Primer set	
	**Forward primer (5'-3')**	**Reverse primer (5' -3')**
**For gene isolation**		
Ci*NF-YC1*-Full	TACACCCCACTTCACCTTATC	CTGATTACACTATGTATAGCAACTC
Ci*NF-YC2*-Full	AGTTGTATAAACATAACCCAGAACC	GAACCAGGACTTAACATGAGATATC
Ci*NF-YC3*-Full	TTTGAGGTACAGCATTTTCACCC	CGAGTAGACTCGAGGCTTTAGC
Ci*NF-YC4*-Full	ACAGATACTTTTTTTCTTCTAGGG	CGTCGTATAACATCATTGAGCATAC
Ci*NF-YC5*-Full	AGTTTGTTTATGTCTCATCATGTTG	TAGTTAATTATTCAACCAACATTCC
**For vector construction of overexpression in *Arabidopsis***		
Ci*NF-YC1*-OX	ACCAAATCGACTCTAGTCTAGA	GTCTTTGTAGTCCATGGTACC
	ATGGACAACAACACAACACCAA	CCCCTGGCCATCGATACC
Ci*NF-YC3*-OX	ACCAAATCGACTCTAGTCTAGA	GTCTTTGTAGTCCATGGTACC
	ATGGATCAGGAAGGGCATG	AGCATCTTCTGTGGTTCTTTTGT
vector-Super1300	GCCATTTCGCCTTTTCAG	CGGTCTTGCGATGATTATCA
**For RT-qPCR analysis**		
Ci*NF-YC1*	CCCACGGGAATGTATGGTCC	GCCACTCCCAAATGACCCAT
Ci*NF-YC2*	ACATGGCACCTCAGCATTCA	ATATATGGCGGAGGCTGCTG
Ci*NF-YC3*	GCTGTTGGTAGCTCTGCTCA	GTTGTGCTGGTGGAGACGTA
Ci*NF-YC4*	GGCAGTCACAGAACATGGGA	GAAGGAAGACCCACCGACTG
Ci*NF-YC5*	TCAGGAGGAGGGATATCGGG	ACACAAGTGGTGCCTCTCCT
Ci*Actin*	TCCGTTGCCCTGAGGTTCT	GATTTCCTTGCTCATCCTGTCA
At*LEAFY*	CAACGGAGGTAGTGGTTTGG	GCTAATACCGCCAACTAAAGC
At*GI*	TGCATCTGGTGTAAGGCTACC	CCTATAGCCCGCAAGAAGTG
At*CO*	GCCTACTTGTGCATGAGCTG	GTTTATGGCGGGAAGCAAC
At*SOC1*	AACAACTCGAAGCTTCTAAACGTAA	CCTCGATTGAGCATGTTCCT
At*FT*	CTGGAACAACCTTTGGTGAG	TACACTGTTTGCCTGCCAAG

At*FLC*	GGCTAGCCAGATGGAGAATAATCA	CCGCCGATTTAAGGTGGCTA
At*SPL3*	CTTAGCTGGACACAACGAGAGAAGGC	GAGAAACAGACAGAGACACAGAGGA
At*SPL4*	TCAAGGGTAGAGATGACACTTCCTATGC	TCTCTCATCATAGCAAGTGATGGACCCTG
At*SPL5*	CCAGACTCAAGAAAGAAACAGGGTAGACAG	TCCGTGTAGGATTTAATACCATGACC
At*SVP*	GCAACTAACGGAAGAGAACGAG	GAGCTCTCGGAGTCAACAGG
At*GA20ox1*	AGATTACTTCTGCGATGCGTTGG	TCTTGATACACCTTCCCAAATGGC
At*GA20ox2*	CAAGAGTTCGAGCAGTTTGGGAAG	TCGGAAATAGTCTCGGTTTACGC
At*GA20ox3*	ACATAGGCGACACCTTCATGGC	TCCTTTCTCTCTCGCTGTTCACC
At*GA20ox4*	GAACATTGGCGACACTTTAATGGC	TGGTGGCTTCACCACTTTGTCC
At*GA20ox5*	AACGTTGGAGACACCTTCATGGC	ACTGCCCTGTGGTAACAACTCC
At*Actin*	TGGGATGAACCAGAAGGATG	AAGAATACCTCTCTTGGATTGTGC
