# Supplementary material for: Genome-Wide Characterization of Chrysanthemum indicum Nuclear Factor Y, Subunit C Gene Family Reveals the Roles of CiNF-YCs in Flowering Regulation
Source: Int J Mol Sci. 2022 Oct 24;23(21):12812. doi: 10.3390/ijms232112812 (PMC9654237; doi:10.3390/ijms232112812)
Supplement: Supplementary file 1 [file ijms-23-12812-s001.zip › supplementary data S1.genomic sequences of CiNF-YCs .pdf]

Supplementary Data S1. The genomic sequences of CiNF-YC

>CiNF-YC1

TACACCCCACTTCACCTTATCCTCTCCCAAAACACATGCATTCTATTCTTATTTTCTACAATCCATCACTTTATTATT
CATATTGGCCTTAACATAACAACCACAAACAACAACCTTAACAACCACAAACAACAACA**ATGGACAACAACACA**
ACACCAACAACAGCCACCCCAACCACCCCTCCGCCACCACCTTACCAACATCTCCTCCAACAACAACACCAA
CAGCTCCAAATGTTTTGGAACTACCAACGTCAAGAAATCGAACAAGTTAATGATTTCAAAAACCATCAACTC
CCTTTAGCACGTATTAAAAAGATCATGAAAGCCGACGAAGACGTTCGCATGATTTCAGCCGAGGCACCTAT
CTTGTTTGCGAAAGCATGCGAGTTATTCATTCTTGAGTTAACTATACGTTCATGGCTGCATGCAGAAGAAAA
TAAACGTAGAACATTGCAAAAAAATGATATTGCAGCTGCTATTACACGTACGGACATATTTGATTTCCTTGT
GGATATTGTTCCACGTGACGAGATTAAGGATGATGTTGGTGTTGGTGTTGGTGTTGGGGGGATGGTAGGGT
CTAATACGGCTAGTGGGATGCCGTATTATTATCCTCCCATGGGACAACCGGCCGCTGGGATGGGTGGGGTG
ACCATGGGACGGCCGGCTGTGGATCCCACGGGAATGTATGGTCCCCCGTCTCAAGCGTGGCAGTCAGTGTG
GCAGACCGGGGATGATGGGTCATTTGGGAGTGGCGGGAGTGGTGGACATGGAGGTATCGATGGCCAGGGG
TAAAGGCCTATTCCTTGATTCGGGCCAATGAGTTCGATATATCAGGGAGGGGCGGGGCGGCGTAGAAGTAGCAGC
TGCCTGCTGGTGAAGATCAGATTGCTCATGGAGTAATTCCTGCAAATTATGATATGAAGTTGGAGTTGCTATACAT
AGTGTAATCAG

>CiNF-YC2

AGTTGTATAAACATAACCCAGAACCAATTACATAAATATCGAGCAAG**ATGGATCCACA**
AGGGCACGGGCAGCCCCAAGGTAGCCACATCATGACCAATGTACCTCAGTTACCA
TATGCAACAAACCCATATGATCAAACCCAAATAAGTGGCACCCCTGGCAGAGGAC
CAATAGTACCACCAGGTGGAGCTGCCCTTCAAGCCGCTACTCAGCCCACTGCAG
CGCAATTAGCACAACAACAAATGGCTTATCATCAGATACAACAACAACAGCAGCA
GCAACTTCAGCAACAACTCCAGTCATTTTGGGCAAACCAGTATCGAGAAATAGAA
AACGTCAATGATTTCAAGAACCATAGCCTTCCATTAGCAAGAATTAAGAAGATCAT
GAAGGCTGACGAGGACGTAAGAATGATATCAGCTGAAGCACCTATTGTGTTTGCA
AGAGCATGTGAGATGTTCATATTGGAGTTGACTCTACGGTCATGGAATCATACTG
AAGAAAACAAAAGGAGGACACTGCAAAAAAACGACATTGCTGCTGCAATCACGA
GGACTGAAATATTTGACTTTTTGGTTGATATAGTTCCGAGGGAGGATATAAAAGAA
GAAGTTATGACATCTATGCCAACGGTGAATGTGCCGGTTGGTGGGCCTTCTGATA
CGTTCCCTTATTATTACATGGCACCTCAGCATTCACCGCAGTCCGGAGCACCAGG
GATGATTTCAAACAAGCCTATGATGGATCCTGGTCTTTATGCACAGCAGCCTCCG
CCATATATGGGTCCAGCAATGTGGCCACAGCCACCTCAGCAGCCACAGTCACCTT
CGGATTCATAGATGCTATAGCCTATTCCTTCTTTGCAAATTTTGGAACTGCTTTGAATCT
ATAATCTGCGTTTGACTCAACTAGGAGTTTATCCGGAACGGGTGGTGTTGGATATCTCA
TGTTAAGTCCTGGTTC

>CiNF-YC3

TTTGAGGTACAGCATTTTCACCCAATTTCCAATCAAACCCTAACCCTAAAAAGCTACTGAAATCATCATCAAGTTC

TTGATTAAATTTTTTGCTCTGGTTCTCAGTTTGATTCAAACATATTAGTTCTTCAAAGGTGTGAGCGTTATTAAGAT
GGATCAGCAAGGGC**ATGGATCAGCAAGGGCATGGGCAAGCCTCAGGTATGGGGGCTGTTGGTAGCTCTGCT**
CAGATGCCTTACAATATGGCACCCTATCCTCCAAACCAAATGATGGGGATTGTACCCCCTGCATCAGTTGGA
GGGTCGGTCCCACCTTCACAAACATCTGGTCTTACGTCTCCACCAGCACAGCTTGCACAACAGCAACTCGCT
TATCAGCACATTCACCAGCAACAGCAACAACAGTTGCATCAACAACTGCAAAATTTCTGGGCAAACCAGTAC
CAAGAAATCGAGCAAACCACTGATTTCAAGAACCATAGTTTACCCTTGGCTAGAATCAAGAAAATCATGAAA
GCAGATGAGGATGTACGAATGATATCTGCTGAAGCACCCGTCATATTTGCTCGTGCATGTGAAATGTTCATC
CTTGAGTTGACTTTAAGGTCTTGGAATCATACTGAAGAGAACAAAAGGAGGACACTTCAGAAAAACGACAT
TGCTGCTGCAATCACAAGGACTGATATCTTTGACTTCTTAGTTGATATTGTACCAAGAGAGGATCTGAAAGA
TGAAATTATTGCTTCTACTGTCCCAAGAGGCGGACCACTTCCAGTTGGAGCTCCAACCGAGGGTCTTCCGTA
TTACTATATGCCTCCTCCACAAGTTGGTGCTTCAGGGATGTATATGGGTAAGCCTGTGGATCCTCAAGCTCT
TTATGGACAGCAGCCTCGTCCATACATGGCTCAGCCTATGTGGCCACAACAGCAACAACAAGAACCACA.GG
AAGATGCTTAAACAGGACGTTTACAAGGGATCAAATCCTGAAGATGGTGGCTGTAGACGAGTACTCACTTCTGGC
ATTTGTATCAAGGTTCAATTTGCATTTGGTTATTAGGAATGCTTGTAGTCATACTTAACTATGTATTTCTGTCTACTT
GCTGTTTAATGATGTTGGCGGCGAACGTGTGTTATATCTAGACCTGGTTCTTGAAAACTTAAACTCATATCCAATG
CAAAAGGTATGGTGATTGCTCTTTCGATGGCAATAAATTAGCCAACACACAACTTTTGAGTGCTAAAGCCTCGAGT
CTACTCG

>CiNF-YC4

ACAGATACTTTTTTTTCTTCTAGGGTTTAATCGAAGTAATTTAAAAGTTTTAAGAGAATT
TGTTGGAAATATCTAAAC**ATGGATCAACAAGACCAGGGGCAGTCACAGAACATGGG**
AACAGCTGGTAGCTCAGCTCAGATGCAATATGGTGTAAGCCCATATCCACAAAAC
CAGATGATGGGCTCCGGGCCCCCAGGTCAGGCTCAACAATCGGTGGGTCTTCCT
TCACCTCCAGGACAGCTTGCACAACAGCAACTTGCTTATCAGCACATTCATCAGC
AACAACAGCAGCAACTGCATCAACAACTTCAGAATTTTTGGGCGAATCAGTACCA
AGAAATCGAGCAGACAACTGATTTTAAAAACCATAGTCTACCATTGGCAAGGATA
AAAAAGATCATGAAGGCAGACGAGGATGTGAGGATGATATCAGCTGAAGCCCCT
GTCATATTTGCTCGTGCATGTGAAATGTTTATACTAGAGTTGACTTTAAGGTCATG
GAATCATACTGAAGAGAATAAACGAAGAACACTTCAGAAAAACGACATTGCAGCT
GCAATCACGAGGACTGATATTTTCGACTTTCTAGTTGATATTGTACCAAGGGAGGA
TTTAAAAGATGAGGTGCTCGCATCAACGATGCCTAGAGGTGGGCCTCTCCCAGTT
GGCGGCCCAACTGAGGGTCTCCCTTACTATTACATGCCTCCGCCACAAGTTGGTG
GTTCAGGGATGTATATGGGTAAGCCCGTTGATCCTCAAGCTCTTTATGGACAGCA
GCCTAATCCATATATGGCTCAGCCTCTTTGGCCACAACAGCAGCAACAAGAACCG
CAGGGTGATGCTTAGGGCAGGGACCTAAAAG.AAGCTTTAGGTAATGTAACAATAGG
GATGTATCAAGCTACAAAAGATCATACTATGGTTGTAATATCTTGAAGACACTCGGTGTA
TGCTCAATGATGTTATACGACG

>CiNF-YC5

AGTTTGTTTATGTCTCATCATGTTGATCTTATTAATATATTCTTCATTACATCATCTTCTCT
CTTAAAATCTTTTGAAAAATAAGCTTTTATGATTTTTGCCCTAGAAATACATGCTTCTTT

GTTCATGAAATATTGATACTAGTGACTTTAGTATAGAACAAAAGTTTTTCTTTGAAACCA
TCAATTGTTTCTTGAACCATCGATTCAGACTATTTCGTTTTCTTTTTATATTTGAG**ATGAG**
GCAAGCCGGGGCGTATTCAGGAATTTTATCAGGAGGAGGGATATCGGGGCGAAC
TGGACCCCATTTGTTGCCATTAGCAAGGATCAAGAAGATCATGAAGAAATCAAGT
GTGGATGTGAAGATGATCTCAGGAGAGGCACCACTTGTGTTCTCAAAAGCTTGT
GAGCTTTTCATTGAGGAATTGACTAAGAGATCATGGAACATGACAATTCAAGGCA
AGAGAAGAACTTTGCACAAAGAAGATGTTGCATCCGCGGTTGCAACGACTGATG
TCTTTGATTTTCTTATCGACTTGGTTCATCCTAATGTTTCTGAAGATGTTGATTTG
AATGATGAAGAAACCCTTGAAAAGTAACTTGAACGCGCGAGCCATGCATTGTTGTTA
ATGAAGTGATTAAAACTTGTTTAACTATGAATATCTCAAAGTCTTGGGATCATATCTTTA
ATTAATCGTGGAATGTTGGTTGAATAATTAACTA
