# Supplementary figures and images for: Genome-Wide Characterization of Chrysanthemum indicum Nuclear Factor Y, Subunit C Gene Family Reveals the Roles of CiNF-YCs in Flowering Regulation
Source: Int J Mol Sci. 2022 Oct 24;23(21):12812. doi: 10.3390/ijms232112812 (PMC9654237; doi:10.3390/ijms232112812)

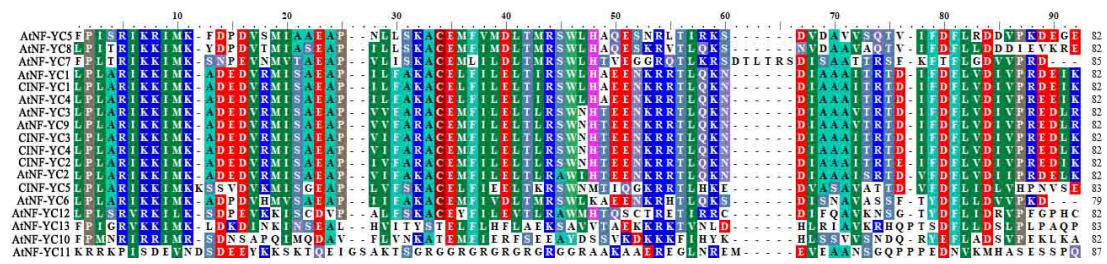

Supplementary Figure S1. The alignment of conserved domain of AtNF-YCs and CiNF-YCs.

Supplement: Supplementary file 1 [file ijms-23-12812-s001.zip › Supplementary Figure S1. The alignment of conserved domain of AtNF-YCs and CiNF-YCs..pdf]

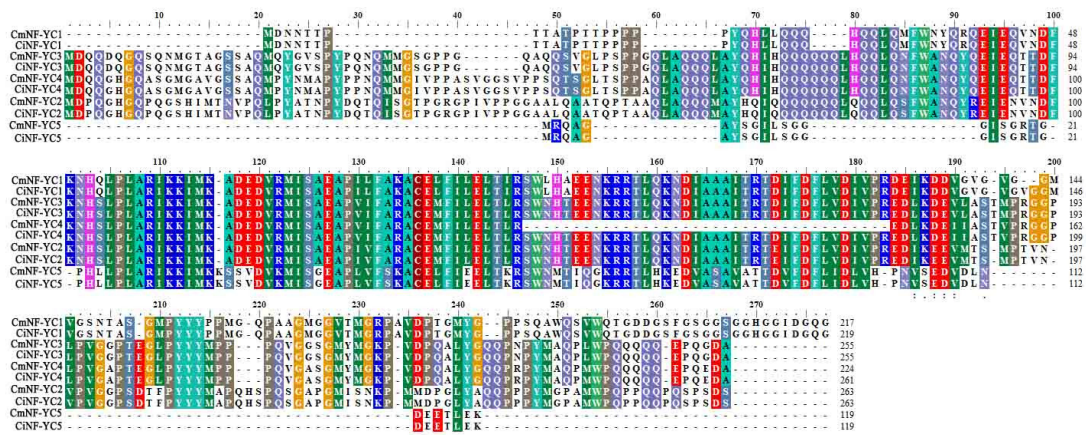

Supplementary Figure S2. The alignment of conserved domain of CmNF-YCs and CiNF-YCs.

Supplement: Supplementary file 1 [file ijms-23-12812-s001.zip › Supplementary Figure S2. The alignment of conserved domain of CmNF-YCs and CiNF-YCs..pdf]

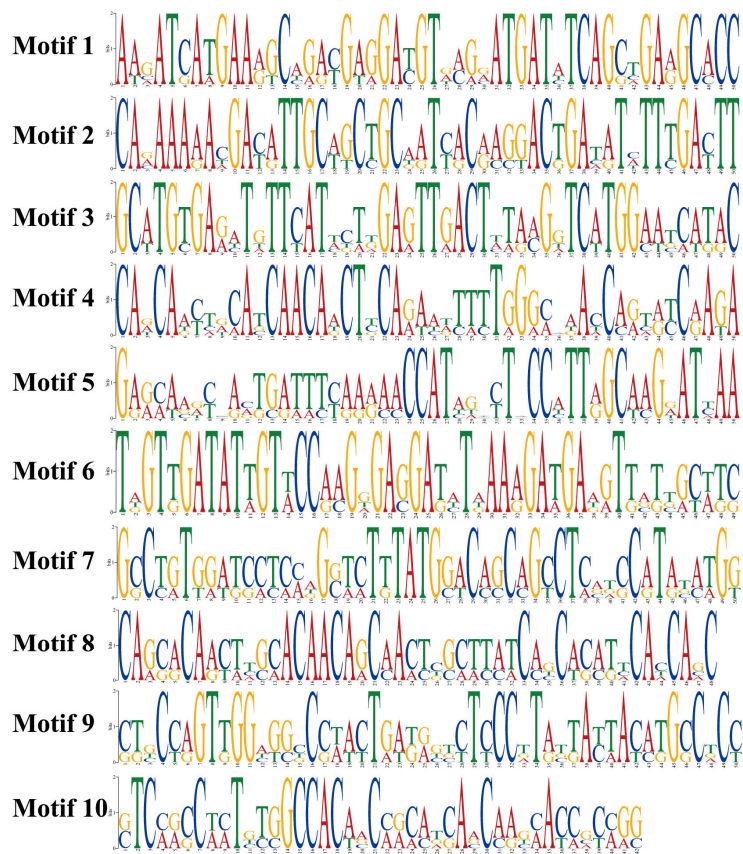

Supplementary Figure S3. The conserved motifs of CiNF-YCs.

Supplement: Supplementary file 1 [file ijms-23-12812-s001.zip › Supplementary Figure S3. The conserved motifs of CiNF-YCs..pdf]
